# High-dose-rate vaginal brachytherapy for treatment of recurrent high-grade vaginal intraepithelial neoplasia

**DOI:** 10.1007/s11547-026-02208-x

**Published:** 2026-03-31

**Authors:** Jacopo Di Muzio, Niccolò Gallio, Valeria Chiofalo, Mario Preti, Fulvio Borella, Elena Casetta, Stefano Cosma, Alberto Revelli, Paola Cassoni, Umberto Ricardi

**Affiliations:** 1 Department of Oncology “AOU Città Della Salute E Della Scienza Di Torino”, Radiotherapy Unit S. Anna Hospital, 10126 Turin, Italy; 2 Department of Surgical Sciences Gynecology and Obstetrics 2U “AOU Città Della Salute E Della Scienza Di Torino”, University of Turin, 10126 Turin, Italy; 3 Department of Surgical Sciences Gynecology and Obstetrics 1U“AOU Città Della Salute E Della Scienza Di Torino”, University of Turin, 10126 Turin, Italy; 4 Department of Medical Sciences Pathology Unit “AOU Città Della Salute E Della Scienza Di Torino”, University of Turin, 10126 Turin, Italy; 5 Department of Oncology Division of Radiation Oncology “AOU Città Della Salute E Della Scienza Di Torino”, University of Turin, 10126 Turin, Italy

**Keywords:** High-grade vaginal intraepithelial neoplasia, VaIN, Brachytherapy, Treatment, High-dose-rate vaginal brachytherapy, Recurrence

## Abstract

**Purpose:**

High-grade vaginal intraepithelial neoplasia (HG VaIN) is a rare preinvasive lesion characterized by a high risk of recurrence. Management strategies encompass both surgical excision, ablative procedures, and topical treatments. Brachytherapy provides a less invasive option, particularly for recurrent HG VaIN cases where other therapies have been unsuccessful or are not feasible. The present study assessed effectiveness and safety of brachytherapy in treating HG VaIN, with a focus on long-term outcomes, including local disease control, progression to invasive carcinoma, and treatment-associated adverse effects.

**Material and methods:**

A single-center retrospective analysis was performed on 31 patients with histologically proven HG VaIN who underwent brachytherapy at St. Anna University Hospital in Turin, Italy, from 1997 to 2024. High-dose-rate vaginal brachytherapy (HDR-VBT) was delivered, with dosage and fractionation schedules customized based on the characteristics of the lesions and individual patient factors.

**Results:**

The median age of the patients was 65 years, with 87.1% having a history of hysterectomy and 96.78% being postmenopausal. All patients had undergone multiple prior treatments (average of 3.7). Total radiation doses varied between 30 and 50 Gy, delivered across 5–10 sessions. Following a median follow-up period of 46.9 months, the local control rate at both 5–10 years was 96.8%, with no cases progressing to invasive carcinoma. Adverse effects were generally mild, with only one grade 3 event.

**Conclusion:**

HDR-VBT provides excellent local disease control with low toxicity in patients with HG VaIN who have received prior treatments.

## Introduction

**Fig. 1:**
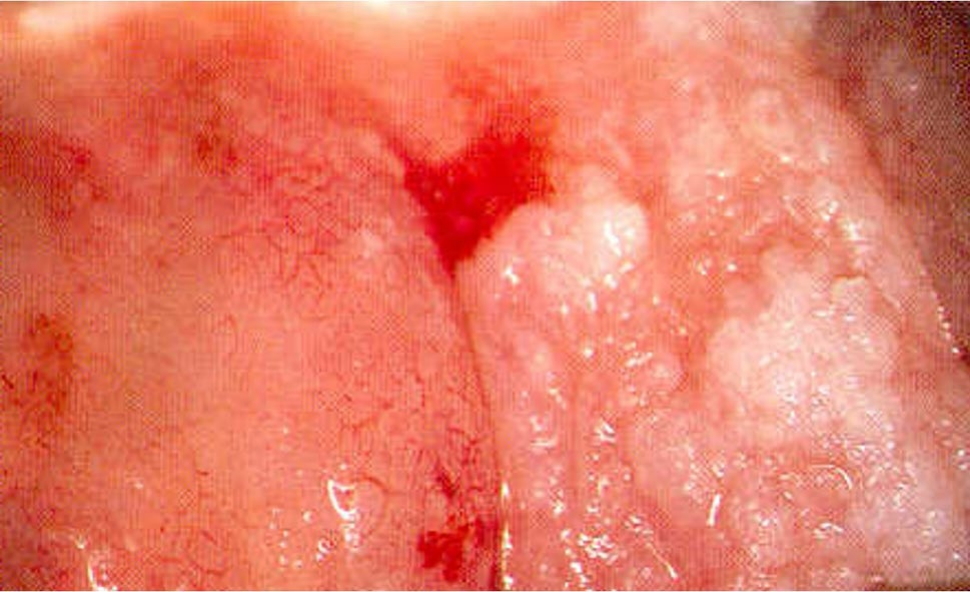
Colposcopic image of high-grade vaginal intraepithelial neoplasia: thick acetowhite epithelium involving vaginal vault with coarse mosaic

Vaginal intraepithelial neoplasia (VaIN) is an uncommon premalignant condition affecting the vaginal epithelium, marked by dysplastic changes that could potentially develop into invasive vaginal cancer if not treated (Fig. 1) [1, 2]. VaIN is closely linked to infection with high-risk strains of human papillomavirus (HPV) and frequently occurs alongside or subsequent to HPV-associated lesions in the cervix, vulva, or anus[3, 4]. Compared to HPV-driven cervical lesions, VaIN is far less prevalent, with an incidence estimated to be approximately 100 times lower[5], resulting in limited research on its optimal treatment strategies.

According to the 2020 WHO classification, VaIN is categorized into a two-tier system: low-grade squamous intraepithelial lesions (LSIL, equivalent to VaIN1) and high-grade squamous intraepithelial lesions (HSIL, including VaIN2 and VaIN3) [6]. Low-grade VaIN typically reflects an active HPV infection with a strong likelihood of resolving spontaneously[7]. In contrast, vaginal HSIL is considered a precancerous state, carrying an estimated 10% risk of progressing to invasive cancer[7, 8].

Managing VaIN involves balancing effective intervention with the preservation of vaginal anatomy and function, particularly in postmenopausal patients or those with prior gynecological surgeries [9]. Due to the absence of robust evidence-based guidelines, several professional organizations, such as the European Society of Gynecological Oncology (ESGO), the International Society for the Study of Vulvovaginal Disease (ISSVD), the European College for the Study of Vulval Disease (ECSVD), and the European Federation for Colposcopy (EFC), have developed consensus-driven recommendations for managing these lesions [10, 11].

Treatment approaches for VaIN have advanced, moving from conventional surgical excision to modern techniques like CO₂ laser ablation or excision[10, 11]. Alternative options, such as topical imiquimod, are also considered, while 5-fluorouracil is now deemed outdated[12, 13]. Typically, therapeutic intervention is reserved for high-grade VaIN (VaIN2 or VaIN3), whereas low-grade lesions (VaIN1) are often managed conservatively due to their tendency to regress without treatment [7, 14, 15].

In this context, brachytherapy has emerged as a minimally invasive treatment option for high-grade VaIN, particularly for multifocal lesions, recurrent disease after surgery, cases unsuitable for local ablation, or patients with medical comorbidities [16]. By delivering targeted high-dose radiation to the affected area, brachytherapy ensures effective local control while minimizing damage to surrounding healthy tissues, such as the bladder and rectum. This is particularly beneficial for patients with a history of pelvic radiation or surgical interventions, which may restrict other treatment options[17].

The incorporation of brachytherapy into the management of VaIN represents a valuable alternative or complement to traditional approaches[10, 11]. High-dose-rate vaginal brachytherapy (HDR-VBT) offers adaptable treatment planning, allowing precise dose adjustments based on the lesion’s location and extent[16, 17]. However, additional studies are required to develop standardized protocols, refine dosimetry, and evaluate long-term outcomes, including recurrence rates and impacts on quality of life.

Despite its advantages, brachytherapy poses clinical challenges, such as the need for tailored planning to account for vaginal anatomical variations, lesion characteristics, and prior treatment histories. Advanced imaging techniques are essential to optimize therapeutic outcomes.

This study presents a retrospective analysis of patients with high-grade VaIN treated with HDR-VBT, offering insights into the application of brachytherapy in this context, including its safety profile and survival outcomes.

## Materials and methods

A monocentric retrospective cohort study was conducted at University Hospital St. Anna, Torino, Italy, from 1997 to 2024. Patients diagnosed with histologically confirmed VaIN 2–3 or vaginal HSIL and treated with brachytherapy were included.

All patient records were retrospectively identified from a dedicated institutional database. Clinical and pathological information extracted from medical files included: age at the time of diagnosis, parity, menopausal status, documented history or concurrent diagnosis of cervical or vulvar lesions confirmed by histology, rationale for any hysterectomy performed, number of previous treatments, HDR-VBT treatment details, follow-up visits, and type of recurrence.

VaIN diagnosis was established histologically, in accordance with the nomenclature of the World Health Organization (WHO)[6] and the Lower Anogenital Squamous Terminology (LAST)[18].

## Treatment protocol

Patients underwent HDR-VBT based on clinical and logistical considerations. HDR-VBT was delivered using single-channel or multichannel vaginal applicator (Nucletron Elekta, The Netherlands), and the choice was based on clinical and colposcopic presentation. All the patients underwent clinical examination and colposcopy with both a gynecologist and a radiation oncologist. Pre-treatment diagnostic imaging with magnetic resonance and/or computed tomography scan was performed to rule out invasive disease.

The delivered dose ranged from 13 to 50 Gy in 2–10 fractions with a range of Equivalent 2 Gy Dose (EQD2) from 62.5 to 17.8 Gy and a Biological Equivalent Dose (BED) range from 75 to 21.5 Gy. All the patients underwent a planning imaging with the applicator in place. From 1997 to 2010, this imaging was represented by 2D antero-posterior and lateral radiographs, then from 2011 to 2024, we adopted a computed tomography scan (3D brachytherapy).

To minimize side effects, patients were planned with a full bladder and empty rectum, and in case of 2D imaging, insertion of a Foley catheter containing a water-contrast solution and a rectal probe with radiopaque markers was performed. In case of 2D brachytherapy, treatment volumes were defined based on geometric assumption using radiographs and ICRU Report 38 guidelines. Doses were prescribed at high-risk clinical target volume, defined as the vaginal wall at 5 mm depth from the applicator surface and eventually at the cervical surface. Plans were calculated with a 2D planning system (Plato–Nucleotron–Holland) for 2D imaging.

**Fig. 2:**
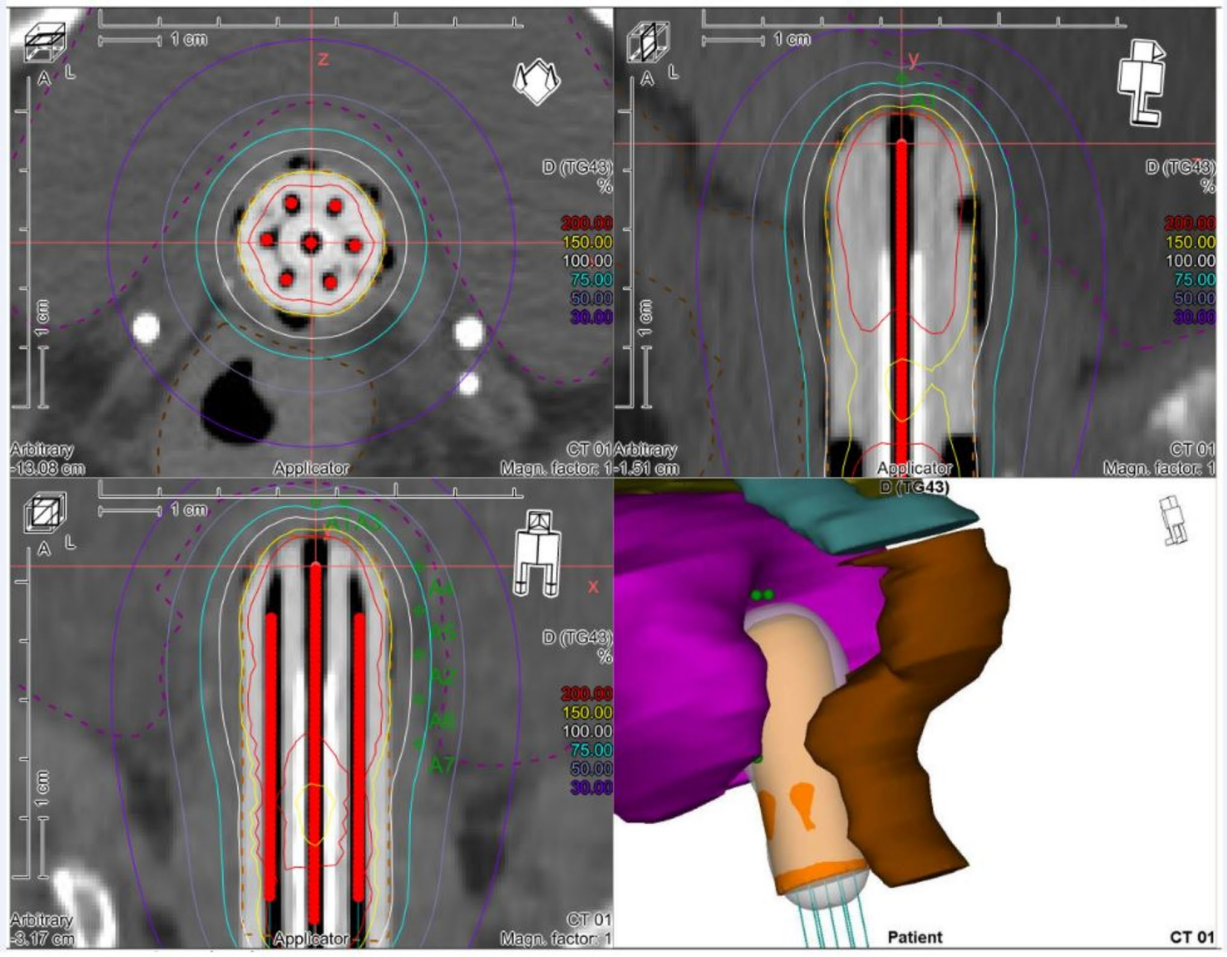
Dose distribution planning

In 3D brachytherapy, treatment volumes were defined using the computed tomography, allowing for image-guided contouring of target volumes and organs at risk (OARs, Fig. 2). The high-risk clinical target volume was therefore delineated based on the computed tomography (integrating magnetic resonance when available, and/or clinical exam data—according to **GEC-ESTRO/ICRU 89 guidelines**) and anatomical extent. A 5 mm depth from and eventually at the cervical surface was still considered when defining coverage and dose prescription. Plans were calculated with a 3D planning system (OncentraBrachy, Nucleotron Elekta, The Netherlands).

Dose-volume histograms were reviewed to ensure compliance with recommended organ-at-risk (OAR) constraints. OARs were defined as bladder, rectum, sigmoid, and bowel; doses at 2 cc were recorded. HDR-VBT was delivered using an iridium-192 (192Ir) source afterloader (Elekta Flexitron—Elekta, Stockholm, Sweden).

## Follow-up

Patients were followed up every 3/4 months for the first year and every 6 months thereafter, with pelvic examinations, cytology, and imaging as clinically indicated. Late toxicity was documented and categorized by affected organ system and severity.

## Outcome measures

The primary outcome was disease-free survival (DFS), defined as the resolution of dysplasia on follow-up cytology and/or biopsy at 6–12 months. Secondary outcomes included recurrence rates and treatment-related toxicity (graded according to CTCAE v5.0)[17]. A recurrence was defined as any biopsy-proven vaginal HSIL, while progression was a histologically documented vaginal squamous invasive carcinoma during follow-up.

## Statistical analysis

Descriptive statistics were used to summarize patient demographics, lesion characteristics, and HDR-VBT details. Treatment efficacy and recurrence rates were analyzed using the Kaplan–Meier method for survival analysis. All statistical analyses were performed using IBM SPSS Statistics, version 29 (IBM Corp., Armonk, NY, USA).

## Results

Clinical and treatment characteristics are summarized in Table 1.

**Table 1:** Clinical and treatment characteristics of the whole cohort

Characteristics	
*Mean Age at treatment (range)*	65 years (44–90**)**
*Hysterectomy*	87.1% (27)
Reason for hysterectomy	
Invasive cervical carcinoma	51.8% (14)
Persistent cervical HSIL	44.4% (12)
Benign disease	3.8% (1)
*Mean time from hysterectomy to VBT*	73.1 months (3–312 months)
*Previous radiation therapy on the pelvis*	6.5% (2)
*Biopsy results*	
VaIN 2	12.9% (4)
VaIN 3	87.1% (27)
*HDR-VBT schedules*	
50 Gy in 10 fractions (EQD2 62 Gy)	3
40 Gy in 8 fractions (EQD2 50 Gy)	13
32.5 Gy in 5 fractions (EQD2 44.69 Gy)	13
16 Gy in 4 fractions (reirradiation) (EQD2 18.67 Gy)	1
13 Gy in 2 fractions (reirradiation) (EQD2 17.88 Gy)	1
*Length of vagina irradiated*	
2/3 upper vaginal length	77.4% (24)
Total vaginal length	22.6% (7)

*HDR-VBT*: high-dose rate vaginal brachytherapy; *VaIN*: vaginal intraepithelial neoplasia

A total of 31 patients were enrolled in the study, with a mean age of 65 years (range: 44–90 years). All participants presented with persistent or recurrent VaIN3 and had undergone extensive prior treatment, with an average of 3.7 previous interventions per patient (range: 1–8).

The majority (96.78%) were postmenopausal. Of the cohort, 87.1% (27 out of 31) had previously undergone hysterectomy, primarily due to invasive cervical carcinoma (*n* = 14), persistent cervical HSIL (*n* = 12), or benign gynecologic conditions (*n* = 1). Median time from hysterectomy to HDR-VBT treatment was 73.1 months (5–312 months).

Two patients with a prior history of cervical cancer were treated with external beam radiation therapy. One received a total dose of 50.4 Gy delivered in 28 fractions, while the other underwent adjuvant therapy with 57.6 Gy administered in 32 fractions. Subsequently, the same patients received vaginal brachytherapy at doses of 16 Gy (in 4 fractions) and 13 Gy (in 2 fractions), respectively, at 28.78–12.98 months following their initial radiation treatment.

**Fig. 3:**
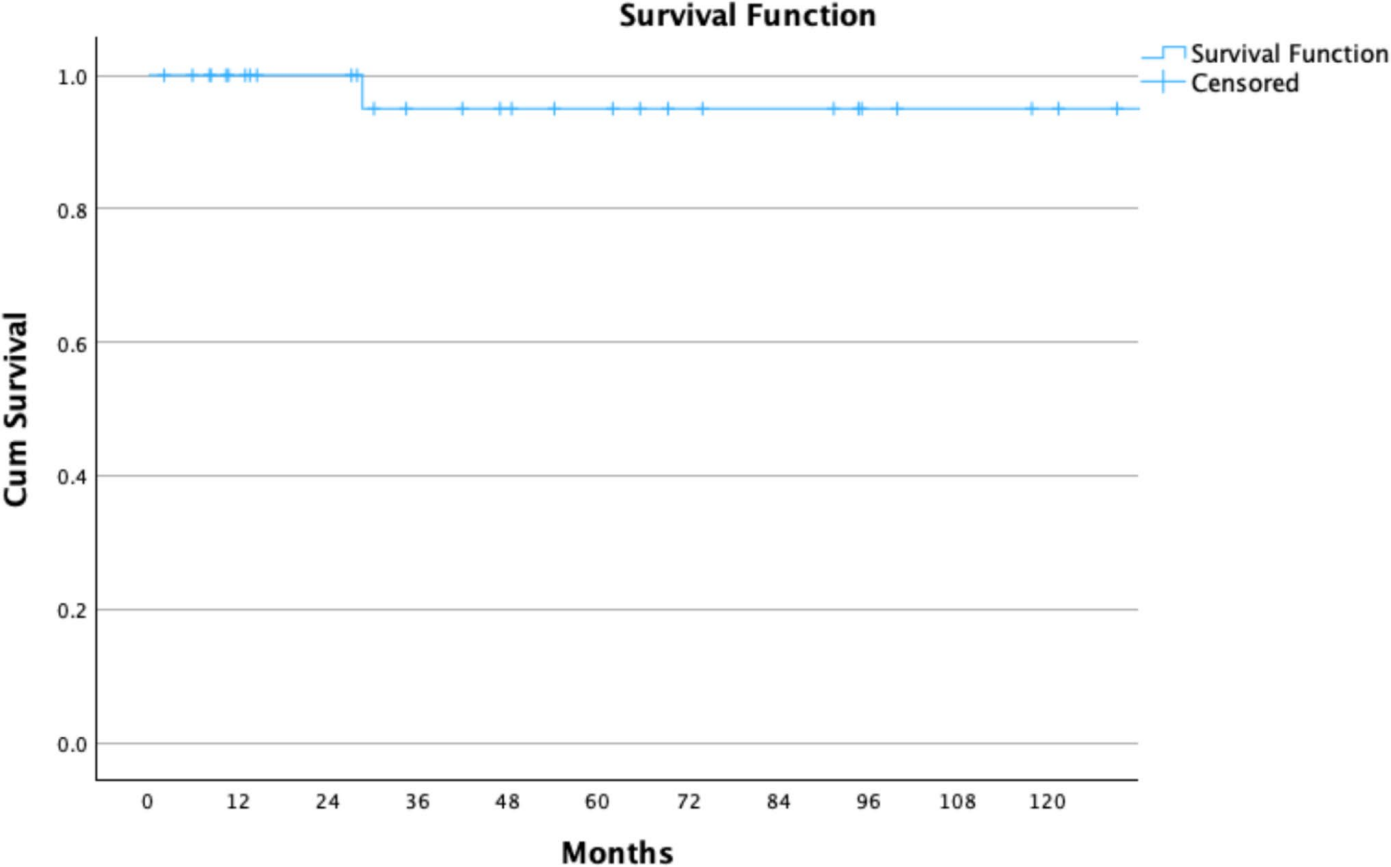
Kaplan–Meier curve for local control

The median follow-up duration was 46.9 months (range: 2.1–133 months). During this period, two patients (6.45%) developed biopsy-confirmed local recurrence of vaginal HSIL at 28.5 and 133 months post-treatment. The 5- and 10-year DFS rates were 96.8% for both observation periods (Fig. 3). Notably, no patient progressed to invasive vaginal carcinoma during follow-up.

Tolerance to HDR-VBT was favorable, with no patients requiring treatment interruption due to adverse effects. Overall, 62.5% of patients experienced treatment-related toxicity, predominantly grade 1–2 (G1–G2).

G1-G2 acute toxicities primarily consisted of radiation-induced cystitis; only one patient (previously treated with radiotherapy for cervical cancer) developed grade 3 (G3) toxicity necessitating temporary treatment discontinuation.

The most commonly observed late adverse event was G1–G2 vaginal stenosis, reported in 62.5% of patients, which was frequently associated with G1–G2 dyspareunia (30%).

## Discussion

The management of High-Grade VaIN remains challenging due to its high recurrence rates and the limited availability of standardized treatment protocols. Brachytherapy has emerged as an effective therapeutic option and a valid alternative to surgery, particularly for patients who are not suitable candidates for surgical excision or those with multiple previous treatments. According to the 2023 consensus statement from ESGO, ISSVD, ECSVD, and EFC for VaIN management, HDR-VBT is recommended in specialized centers, yielding promising outcomes—particularly in patients previously subjected to multiple conservative treatments[10, 11]. The commonly prescribed dose is 60 Gy EQD2 to a depth of 5 mm beneath the vaginal mucosa, with varied fractionation schemes designed to minimize the risk of vaginal stenosis[17, 19–25]. In our clinical practice, the most frequently adopted regimens included 40 Gy delivered in 8 fractions of 5 Gy (EQD2: 50 Gy) and 32.5 Gy administered in 5 fractions of 6.5 Gy (EQD2: 44.7 Gy), both demonstrating high patient compliance over time. The studies summarized in Table 2 show that HDR-VBT achieves high local control rates by delivering targeted radiation. DFS rates ranged from 85,7 to 100%, and our results are in line with those in the literature. Although HDR-VBT is generally well tolerated, treatment-related toxicities—such as mucosal atrophy, fibrosis, and vaginal stenosis—remain clinically relevant concerns, particularly among postmenopausal patients. Acute radiation-induced toxicities are typically mild and most frequently present as cystitis, likely due to the anatomical proximity between the bladder and the vaginal vault. In our cohort, most patients experienced G1 cystitis, which did not require pharmacological intervention. These findings are consistent with previously published data[17]. Only one patient developed G3 cystitis, potentially attributable to prior external beam radiotherapy for cervical cancer. HDR-VBT can lead to long-term structural changes in vaginal tissues, causing direct harm to the mucosa, connective tissue, and microvasculature[26, 27]. These effects may contribute to secondary mucosal atrophy and excessive collagen buildup, ultimately resulting in vaginal shortening and reduced elasticity[28]. Furthermore, abnormal capillary dilation can trigger telangiectasia, increasing the likelihood of bleeding. Adhesions and fibrosis further compromise flexibility and in severe cases, may lead to complete vaginal stenosis. Radiation-induced vaginal complications, particularly within the first two years post-treatment, remain a significant concern. Among the late-onset effects, vaginal stenosis is the most prevalent and often persists over time, making it the primary focus of our toxicity assessment. The extent of epithelial damage is further aggravated by menopausal status, where declining estrogen levels contribute to tissue thinning and reduced lubrication, intensifying discomfort and functional impairment[26, 27, 29]. In our study, most patients (62%) experienced late toxicity, with G2 vaginal stenosis being the most common manifestation. Since this late effect may interfere with colposcopic follow-up, it also raises concerns about the patients' sexual health. Persistent dyspareunia was reported in 32% of our cohort. These findings are consistent with those of Zolciak-Siwinska[16] and Graham[23], who reported G2–3 vaginal stenosis in 35–25% of cases, respectively. The former study also documented dyspareunia in 35% of patients. None of our patients experienced G3–4 late toxicities, although such cases have been reported in the literature [17, 19–25]. Similar to other studies [17, 19–25], our series did not observe any second malignancy following HDR-VBT for VaIN.

**Table 2:** Summary of brachytherapy studies for VaIN treatment

Author/year	Brachytherapy technique	Number of patients included	Histology	Median Follow-up (months)	DFS
Woodman 1988 [18]	Low-dose rate	11	VaIN 3	25	100%
MacLeod 1997 [19]	High-dose rate	14	VaIN 3	46	85.7%
Ogino 1998 [20]	High-dose rate	6	VaIN 3	77.3	100%
Teruya 2002 [22]	High-dose rate	13	CIS	127	100%
Graham 2007 [30]	Medium-dose rate	22	VaIN 3	77	86.4%
Blanchard 2011 [23]	Low-dose rate	28	VaIN 3	41	93%
Song JH 2014 [14]	High-dose rate	34	VaIN	48	88.2%
Zolciak-Siwinska, 2015[16]	High-dose rate	17	VaIN 2/3	39	90%
Barcellini 2019 [24]	High-dose rate	14	VaIN3	NA	NA
Present study	High-dose rate	31	VaIN3	46.9	96.8%

*CIS*: carcinoma in situ; *DFS*: disease-free survival; *NA*: not available; *VaIN*: vaginal intraepithelial neoplasia

## Strengths and limitations

This study analyzed a relatively small cohort of patients with HG VaIN treated with HDR-VBT, allowing for a purely observational and descriptive analysis. Due to the extended study period (1997–2024) and the heterogeneity of clinical indications, certain biases must also be considered. Nevertheless, all patients were treated at a referral center by a dedicated multidisciplinary team comprising gynecologic oncologists, specialized pathologists, and experienced radiation oncologists with extensive expertise in HDR-VBT. Notably, this cohort currently represents the largest published series with a long median follow-up.

## Conclusions

HDR-VBT offers a noninvasive and valid alternative to radical surgery, with minimal acute side effects—particularly in patients presenting with extensive vaginal disease for whom total vaginectomy is not feasible. Although the number of cases in this specific setting is limited, our experience suggests that retreatment with HDR-VBT may be a viable option in cases of HG VaIN. Long-term surveillance and individualized follow-up are essential, given the risk of recurrence and the rarity of this condition. Further research is warranted to refine radiation dosing strategies and to investigate combination approaches—such as brachytherapy with topical agents or immunotherapy—that could improve therapeutic efficacy while minimizing adverse effects that may compromise patients’ quality of life.

## Data Availability

Data are available upon reasonable request to the corresponding author.
